# Hypophosphatemia in Pediatric Patients on Continuous Kidney Replacement Therapy–A WE-ROCK Study

**DOI:** 10.21203/rs.3.rs-9636966/v1

**Published:** 2026-05-20

**Authors:** Faizeen Zafar, Katja M Gist, Indrani Sarkar, Javier A. Neyra, Shanti Balani, Eileen A. Ciccia, Jennifer Nhan, Dua Qutob, Brynna Van Wyk, Sarah N Fernandez Lafever, Denise Colosimo, Eleonora Marinari, Todd Jenkins, Huaiyu Zang, Nicholas J. Ollberding, Shina Menon

**Affiliations:** SickKids: The Hospital for Sick Children; University of Colorado Anschutz Medical Campus; Cincinnati Children’s Hospital Medical Center; University of Alabama at Birmingham; University of Minnesota; St Louis Children’s Hospital; University of Minnesota; Weill Cornell Medicine - Qatar; The University of Iowa; Hospital General Universitario Gregorio Marañón: Hospital General Universitario Gregorio Maranon; University Hospital Meyer: Azienda Ospedaliero Universitaria Meyer; Bambino Gesu Pediatric Hospital: Ospedale Pediatrico Bambino Gesu; Cincinnati Children’s Hospital Medical Center; Cincinnati Children’s Hospital Medical Center; Cincinnati Children’s Hospital Medical Center; Stanford University School of Medicine

**Keywords:** continuous kidney replacement therapy, phosphate, hypophosphatemia

## Abstract

**Background:**

Hypophosphatemia is common during critical illness and in patients receiving continuous kidney replacement therapy (CKRT). In this secondary analysis of an international multicenter study, we aimed to evaluate the incidence of hypophosphatemia, associated outcomes, and impact of phosphate-containing CKRT fluids.

**Methods:**

We conducted a multicenter retrospective cohort study of patient < 25 years from the Worldwide Exploration of Renal Replacement Outcomes Collaborative in Kidney Disease (WE-ROCK) who underwent CKRT for acute kidney injury and pathologic fluid accumulation. Hypophosphatemia was defined as serum phosphate < 2.5 mg/dL during the first 7 days of CKRT. Patients were categorized by CKRT fluid type: phosphate-containing (PHOS+) commercial, PHOS+ compounded, or phosphate-free (PHOS−); those who received > 1 fluid type were excluded. Outcomes included 28-day ventilator-, ICU-, and hospital-free days, and ICU and 90-day mortality.

**Results:**

We included 823 patients with a median age of 9.0 years (IQR 1.7–15.3). The majority received PHOS+ fluids (69%; 572/823). The overall incidence of hypophosphatemia was 31% (257/823 patients), occurring more frequently in those receiving PHOS− (91/251, 36%) compared to PHOS+ fluids (166/572, 29%). On adjusted analysis, commercial PHOS+ fluids had lower odds of developing hypophosphatemia than PHOS− fluids (OR 0.62, 95% CI 0.40–0.96; p = 0.03). Hypophosphatemia was not associated with ventilator-, ICU-, or hospital-free days, or mortality after adjusting for confounders.

**Conclusions:**

Hypophosphatemia occurred in approximately one-third of pediatric patients receiving CKRT. Use of PHOS+ CKRT fluids, particularly commercial formulations, was independently associated with a lower incidence of hypophosphatemia, identifying CKRT fluid selection as a potential modifiable practice.

## INTRODUCTION

Hypophosphatemia is common in critically ill children, with reported incidence ranging from 30–70%, depending on the definition used.[1–3] Recognized risk factors include underlying malnutrition, parenteral nutrition, diuretic exposure, and continuous kidney replacement therapy (CKRT). Phosphate plays an essential role in cellular structure, energy metabolism, and multiple critical physiologic processes.[4] Consequently, hypophosphatemia has been associated with adverse outcomes in critical illness, including prolonged intensive care unit (ICU) length of stay and increased duration of mechanical ventilation.[3] Importantly, hypophosphatemia represents a potentially modifiable risk factor, as phosphate supplementation is readily available. With increasing utilization of CKRT in critically ill children, understanding the incidence, determinants, and consequences of hypophosphatemia in this population is increasingly important.

Studies in adult patients receiving CKRT report a high incidence of hypophosphatemia, affecting up to 50% of patients.[5–8] In contrast, pediatric data are limited and heterogeneous. Santiago et al. reported hypophosphatemia, defined using variable age-based cut-offs, in 68% of a cohort of 47 children treated with CKRT.[9] A more recent single-center study of 177 children found a markedly lower incidence of 10% using a definition of serum phosphate < 2.5 mg/dL.[10] In this study, hyperphosphatemia (> 7.4 mg/dL), and not hypophosphatemia, was associated with worse outcomes. Collectively, pediatric literature describing phosphate derangements during CKRT remains sparse and is further complicated by variable definitions and inconsistent outcome assessment.[11]

Patients receiving CKRT are particularly susceptible to hypophosphatemia due to continuous extracorporeal phosphate clearance, especially when phosphate-free dialysate or replacement fluids are used.[12] Several strategies may be employed to mitigate this risk, including intravenous phosphate supplementation, addition of phosphate to commercially available phosphate-free CKRT fluids, and use of commercially available phosphate-containing CKRT fluids. In adult populations, use of phosphate-containing CKRT fluids has been independently associated with lower rates of hypophosphatemia, fewer ventilator days, and shorter ICU length of stay compared with phosphate-free fluids.[5, 8] To date, however, no large multicenter studies have evaluated the incidence, impact, or prevention strategies for hypophosphatemia in critically ill children receiving CKRT.

The Worldwide Exploration of Renal Replacement Outcomes Collaborative in Kidney Disease (WE-ROCK) is a multinational consortium studying the epidemiology, practices, and clinical and patient-centered outcomes of children and young adults receiving CKRT for acute kidney injury (AKI) and pathologic fluid overload.[13] In this planned secondary analysis of the WE-ROCK registry, we sought to describe the incidence of hypophosphatemia during CKRT, identify associated risk factors, and evaluate the impact of phosphate-containing versus phosphate-free CKRT solutions on the development of hypophosphatemia and relevant clinical outcomes.

## METHODS

### Study Design

The protocol and demographics of the original WE-ROCK study have been previously described.[13, 14] Briefly, WE-ROCK is a multicenter collaborative that includes patients aged 0–25 years who received CKRT for AKI or pathologic fluid accumulation from January 1, 2015, to December 31, 2021. Exclusion criteria were as follows: (1) end-stage kidney disease defined as dialysis dependence prior to CKRT initiation; (2) CKRT for a non-AKI/fluid accumulation indication (i.e., toxic ingestion, inborn errors of metabolism, etc.); (3) concurrent receipt of extracorporeal membrane oxygenation. Additionally, these analyses excluded patients who received > 1 type of CKRT fluid during the first 7 days of CKRT. The institutional review board (IRB) approved the collaborative study at Cincinnati Children’s Hospital Medical Center. Each center received approval from its respective IRB or ethics committee with a waiver of informed consent. This study adhered to the Strengthening the Reporting of Observational Studies in Epidemiology (STROBE) guidelines.[15]

### Demographic and CKRT Data

Baseline patient characteristics were collected, including age, sex, reason for ICU admission, and presence of sepsis (defined as the presence of an infection and systemic inflammatory response syndrome within 24 hours of ICU admission). [16] The Pediatric Logistic Organ Dysfunction (PELOD-2) score[17] and need for mechanical ventilation were used to assess patient acuity within 24h of CKRT initiation. Fluid balance was calculated from ICU admission to CKRT initiation using fluid intake and output.[18] Prescribed CKRT dose, CKRT duration, and type of dialysis and replacement fluid were recorded. Centers were categorized into small (≤ 30 ICU beds), medium (31–60 ICU beds), and large (> 60 ICU beds).

### Exposure and Outcomes

CKRT fluid type was the primary exposure variable and was divided into 3 groups: commercially available phosphate-containing (PHOS+) fluids (1 mmol/L), pharmacy-compounded PHOS+ fluids (1–1.5 mmol/L), and phosphate-free fluids (PHOS−). The primary outcome was hypophosphatemia, defined as a minimum phosphate level of < 2.5 mg/dL within the first 7 days of CKRT initiation.[5, 7] Secondary outcomes included 28-day ventilator-, ICU-, and hospital-free days and 90-day mortality. Ventilator-, ICU-, and hospital-free days were defined as the number of days patients were alive and free from mechanical ventilation (in those who were on mechanical ventilation at CKRT initiation) or discharged from the ICU or the hospital, respectively, within 28 days following initiation of CKRT. Patients who died within this 28-day period were assigned a ventilator-, ICU-, or hospital-free day value of zero.

### Statistical analysis

Continuous variables were reported as medians and interquartile ranges (IQRs) and categorical variables were reported as frequencies and percentages. Group comparisons across CKRT fluid types were assessed using Kruskal-Wallis or Wilcoxon rank-sum tests for continuous variables and Pearson’s Chi-squared or Fisher’s exact tests for categorical variables, as appropriate.

To evaluate temporal trends in serum phosphate levels during CKRT among patients receiving PHOS− fluid, an ordinal regression model incorporating restricted cubic splines with four knots (at the 5th, 35th, 65th, and 95th percentiles of CKRT duration) was used to flexibly model the nonlinear relationship between serum phosphate and CKRT duration (days 0–7). This approach allows smooth, continuous modeling of phosphate changes over time while stabilizing estimates at the extremes of the distribution. Robust standard errors were calculated using Huber-White sandwich estimators clustered by hospital size to account for within-group correlation.

A multivariable logistic regression model was fit to assess the association between clinical factors and the risk of hypophosphatemia. The binary outcome was defined as serum phosphate < 2.5 mg/dL (hypophosphatemia) versus ≥ 2.5 mg/dL (reference). Candidate covariates were selected a priori based on prior literature and biological plausibility and included age (years), sepsis at ICU admission, PELOD-2 score at CKRT initiation, CKRT fluid type, dose (mL/kg/hr), and duration (days), and hospital size.

To evaluate whether hypophosphatemia was independently associated with ICU mortality, we performed multivariable logistic regression adjusting for sepsis at ICU admission, PELOD-2 score at CKRT initiation, admission diagnosis, CKRT duration, CKRT fluid type, and primary comorbidities. To explore whether this association differed by fluid type, we included a CKRT fluid type × hypophosphatemia interaction term and performed stratified analyses fitting separate adjusted models within each fluid group.

Missing data were assumed to be missing at random; no imputation was performed. All statistical analyses were conducted using R (version 4.4.0, https://www.r-project.org/). Regression analyses and spline modeling were performed using the rms package (version 6.8.1). A two-tailed p-value < 0.05 was considered statistically significant.[19]

## RESULTS

### Baseline Cohort Characteristics

**Figure 1:**
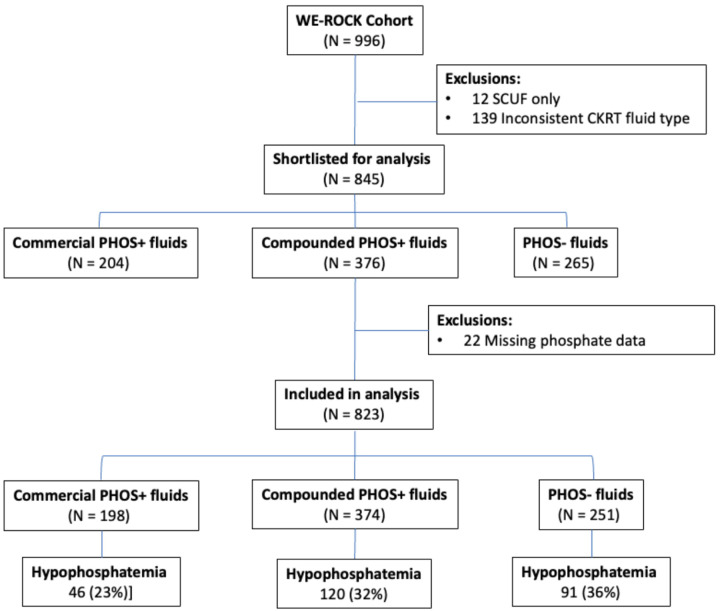
CONSORT Diagram. Hypophosphatemia was defined as <2.5 mg/dl. CKRT, continuous kidney replacement therapy; PHOS+, phosphate-containing; PHOS−, phosphate-free; SCUF, slow continuous ultrafiltration; WE-ROCK, Worldwide Exploration of Renal Replacement Outcomes Collaborative

A total of 823 patients (31 centers, 8 countries) were included in the analysis after excluding those who received slow continuous ultrafiltration only, received > 1 fluid type during the first 7 days of CKRT, or those with missing phosphate levels (Fig. 1). The median age of the cohort was 9.0 years (IQR 1.7–15.3) (Table 1). The predominant admission category was shock, infection, or major trauma (300/823, 36%), with sepsis documented in 360/823 (44%) at ICU admission. The median PELOD-2 score at CKRT initiation was 7 (IQR 4–9). The median fluid balance percentage at CKRT initiation was 7.0% (IQR 2.2–16.8), and the majority of the patients (508/823, 76%) were mechanically ventilated at CKRT initiation.

PHOS+ fluids were used in the majority of patients (572/823, 69%), most commonly as pharmacy-compounded solutions (374/572, 65%) (Table 1). Baseline characteristics and CKRT prescriptions differed across fluid groups. Compared to the other groups, patients receiving pharmacy-compounded PHOS+ fluids were more frequently admitted with sepsis (p < 0.001), had higher median PELOD-2 scores (p < 0.001), and greater percentage fluid overload at CKRT initiation (p = 0.002). Patients receiving PHOS− fluids had a higher rate of mechanical ventilation at CKRT initiation (p = 0.002) and were prescribed higher median CKRT doses (p = 0.03) but had shorter median CKRT duration (p < 0.001).

### Serum Phosphate Trajectory, Incidence of Hypophosphatemia, and Associated Risk Factors

**Figure 2:**
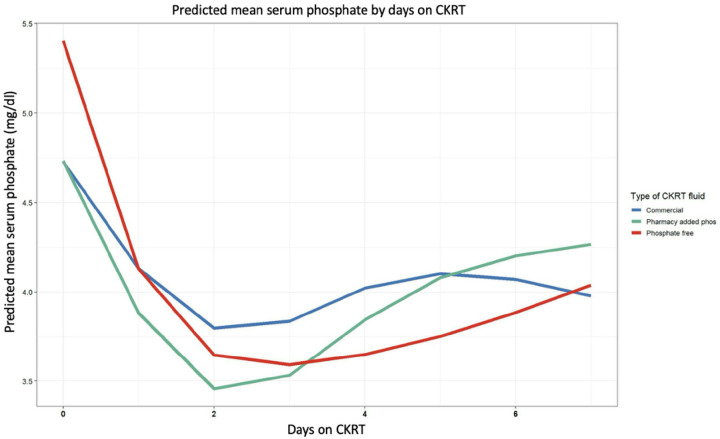
Predicted mean serum phosphate by days on continuous kidney replacement therapy (CKRT) stratified by CKRT fluid type. Across all CKRT fluid types, mean serum phosphate declined after CKRT initiation with an early nadir. The nadir occurred around day 2 in both commercially available and pharmacy-added phosphate-containing fluid groups, whereas patients receiving phosphate-free fluids reached nadir later, around day 3

Figure 2 illustrates CKRT fluid specific serum phosphate trajectories across the 7 day observation period. At CKRT initiation, the median serum phosphate level was 4.7 (3.6–6.0) mg/dL, higher values seen in patients started on PHOS− fluids (p = 0.01). Phosphate levels declined rapidly after CKRT initiation with nadir on day 2 for PHOS+ fluids and on day 3 for PHOS− fluids. Predicted mean phosphate level was the lowest for the pharmacy-compounded PHOS+ group at 2.8 mg/dL. The overall incidence of hypophosphatemia during CKRT was 257/823 (31%), with the highest incidence noted in the PHOS− group (91/251, 36%; p = 0.01) (Table 2).

Tables 3 and 4 show unadjusted and adjusted analyses, respectively, for associations of hypophosphatemia. CKRT fluid choice was an independent predictor of hypophosphatemia, with commercially available PHOS+ fluids being associated with lower odds of hypophosphatemia (OR 0.62, 95% CI 0.40–0.96; p = 0.03) (Table 4). While illness severity and CKRT duration predicted hypophosphatemia risk on unadjusted analysis, they lost significance after adjusting for covariates. Smaller centers had a higher risk of hypophosphatemia when compared to medium-sized but not large-sized centers.

### Impact of Hypophosphatemia on Secondary Outcomes

We did not observe an association between hypophosphatemia and ventilator-free, ICU-free, or hospital-free days (Table 5). Unadjusted mortality was lower among patients who developed hypophosphatemia; however, this lost significance after adjusting for admission category, presence of comorbidities, and illness severity and other covariates in Table 6.

## DISCUSSION

In our large multicenter cohort of pediatric and young adult patients receiving CKRT, hypophosphatemia was a common complication, occurring in approximately one-third of patients. We also noted that phosphate-containing CKRT solutions, specifically commercially available formulations, reduced the incidence of hypophosphatemia in this population, identifying a modifiable practice-level intervention during CKRT.

We used a relatively stringent threshold (< 2.5 mg/dL) to define hypophosphatemia, which may identify fewer cases than studies using higher cutoffs.[20] However, the clinical significance of more modest phosphate deficits remains uncertain, with inconsistent associations reported in the 2.5–3 mg/dL range.[1, 3] The observed incidence of hypophosphatemia was comparable to prior reports.[2] The primary determinant of hypophosphatemia in our cohort was the choice of CKRT fluid, with commercially available PHOS+ fluids having the lowest odds of developing hypophosphatemia. Reducing phosphate removal through the use of PHOS+ dialysis and replacement fluids represents the most effective strategy to mitigate hypophosphatemia.[21] Traditionally, this has been achieved through pharmacy-compounded phosphate supplementation, typically 0.8–1.2 mmol/L, which increases pharmacy workload and introduces potential safety concerns.[12, 22] In contrast, commercially available PHOS+ solutions have consistently demonstrated lower hypophosphatemia rates and may improve standardization of phosphate delivery, though cost and availability remain barriers.[6, 23, 24]

Although higher CKRT doses (40 ml/kg/h vs. 25 ml/kg/h) have been associated with hypophosphatemia in prior studies[25], we did not observe an independent association between prescribed dose and hypophosphatemia in our cohort, potentially reflecting mitigation through widespread use of PHOS+ fluids. This may also explain why CKRT duration was not associated with hypophosphatemia on adjusted analyses.

A meta-analysis of 12 observational studies assessing hypophosphatemia and ICU outcomes found an increase in both ICU and hospital length of stay by approximately 2 days.[3] We did not find similar associations using 28-day ICU- and hospital-free days as outcome measures. Although hypophosphatemia was associated with lower ICU and 90-day mortality on unadjusted analyses, adjustment for important confounders negated this association. Existing literature suggests that hypophosphatemia may represent a marker of disease severity rather than an independent mortality risk factor.[3, 26] However, the absence of an association with short-term mortality should not be equated with an absence of clinically meaningful harm in this population. Adequate phosphate availability is essential for skeletal mineralization, neuromuscular function, and normal growth,[4] and validated measures of these longer-term consequences in critically ill children are currently lacking. Mechanistic and longitudinal studies are needed to define which outcomes are most relevant; in the interim, maintaining phosphate homeostasis during CKRT remains a clinically prudent goal.

### Strengths and Limitations

Key strengths of this study include its large sample size and multicenter design, encompassing patients from 31 centers across 8 countries, which enhances generalizability and reflects contemporary international CKRT practices. To our knowledge, this represents the largest pediatric CKRT cohort to date specifically evaluating the impact of CKRT fluid composition on hypophosphatemia.

There are several limitations to our study. First, the WE-ROCK registry includes patients receiving CKRT for AKI and pathologic fluid accumulation only, which may limit generalizability to other contexts associated with hypophosphatemia. Second, CKRT fluid selection was strongly influenced by institutional practice patterns and center size, resulting in indication and structural confounding. This is evident in the minimal use of PHOS− fluids at large centers, the higher baseline phosphate levels in the PHOS− group consistent with intentional fluid selection, and the higher observed risk of hypophosphatemia at smaller versus medium-sized centers–which likely reflects residual practice-pattern confounding rather than a true center-size effect. Additionally, the registry did not capture phosphate supplementation practices, including riders for repletion and phosphate delivered via parenteral nutrition, which vary substantially across institutions and represent unmeasured confounders.

From a methodological standpoint, exclusion of patients receiving multiple fluid types during a CKRT run may have led to underestimation of hypophosphatemia incidence and attenuation of associations. Phosphate data were limited to the first 7 days of CKRT, restricting assessment of hypophosphatemia risk during prolonged CKRT. Finally, the retrospective observational design precludes causal inference despite adjustment for measured confounders.

## CONCLUSION AND FUTURE DIRECTIONS

Hypophosphatemia is a common complication among pediatric and young adult patients receiving CKRT. In this large multicenter cohort, the use of phosphate-containing CKRT fluids, particularly commercially available formulations, was associated with a significantly lower incidence of hypophosphatemia, identifying CKRT fluid selection as a key modifiable practice. These findings support the need for standardized approaches to phosphate management during CKRT.

Future studies should focus on defining optimal phosphate management strategies, including standardized supplementation protocols and consensus guidelines for children receiving CKRT. Comparative evaluations of commercially available phosphate-containing versus pharmacy-compounded fluids, including pharmacoeconomic analyses, are also warranted to inform implementation across diverse healthcare settings.

## Supplementary Files

This is a list of supplementary files associated with this preprint. Click to download.

- WEROCKGraphicalAbstract.jpg
- SupplementSTROBEWEROCK.docx

## Figures and Tables

**Table 1:** Baseline demographics and CKRT characteristics by CKRT fluid type

Variable	Type of CKRT fluid				
	Overall N = 823^a^	Commercial PHOS+ N = 198^a^	Pharmacy compounded PHOS+ N = 374^a^	PHOS-N = 251^a^	p-Value
**Age (years)**	9.0 (1.7–15.3)	8.8 (1.1–15.6)	9.4 (1.7–5.4)	8.5 (2.2–14.7)	0.78^b^
**Sex**					0.56^c^
Male	440 (53%)	105 (53%)	207 (55%)	128 (51%)	
**Admission category**					**<0.001** ^d^
Shock/infection/major trauma	300 (36%)	59 (30%)	148 (40%)	93 (37%)	
Respiratory failure	153 (19%)	41 (21%)	75 (20%)	37 (15%)	
Post-surgical/minor trauma	41 (5%)	9 (5%)	20 (5%)	12 (5%)	
CNS dysfunction	33 (4%)	6 (3%)	19 (5%)	8 (3%)	
Pain/sedation management	8 (1%)	4 (2%)	2 (1%)	2 (1%)	
Cardiac	99 (12%)	32 (16%)	45 (12%)	22 (9%)	
Other	189 (23%)	47 (24%)	65 (17%)	77 (31%)	
**Sepsis at ICU admission**	360 (44%)	70 (35%)	194 (52%)	96 (38%)	**<0.001** ^c^
**Primary comorbidities**					
None	163 (20%)	42 (21%)	72 (19%)	49 (20%)	0.85^c^
Respiratory	105 (13%)	29 (15%)	47 (13%)	29 (12%)	0.61^c^
Cardiac	148 (18%)	40 (20%)	64 (17%)	44 (18%)	0.64^c^
Neurologic/neuromuscular	98 (12%)	28 (14%)	33 (8.8%)	37 (15%)	**0.044** ^c^
Nephrologic/urologic	74 (9%)	17 (8.6%)	37 (9.9%)	20 (8.0%)	0.69^c^
Hematologic	103 (13%)	15 (7.6%)	28 (7.5%)	60 (24%)	**< 0.001** ^c^
Oncologic	196 (24%)	44 (22%)	102 (27%)	50 (20%)	0.089^c^
Immunologic	121 (15%)	37 (19%)	55 (15%)	29 (12%)	0.11^c^
Gastrointestinal	138 (17%)	36 (18%)	59 (16%)	43 (17%)	0.75^c^
Endocrinologic	51 (6.2%)	9 (4.5%)	25 (6.7%)	17 (6.8%)	0.54^c^
**PELOD-2 score at CKRT initiation**	7 (4–9)	6 (4–8)	7 (5, 10)	6 (4, 9)	**<0.001** ^b^
**%Fluid overload at CKRT initiation**	7.0 (2.2–16.6)	7.2 (2.6–16.1)	7.7 (2.7–20.1)	5.3 (1.0–13.1)	**0.002** ^b^
**Mechanical ventilation at CKRT initiation**	508 (76%)	114 (66%)	193 (78%)	201 (80%)	**0.002** ^c^
**CKRT dose in mL/kg/hr**	43 (31–60)	41 (31–67)	41 (30–57)	48 (33–62)	**0.029** ^b^
**CKRT duration (days)**	6 (3–15)	7 (3–17)	8 (4–18)	5 (2–10)	**<0.001** ^b^

CNS, Central nervous system; CKRT, Continuous kidney replacement therapy; ICU, Intensive care unit; PELOD, Pediatric logistic organ dysfunction; PHOS+, Phosphate-containing; PHOS−, Phosphate-free.

a Median (Interquartile range); n (%)

b Kruskal-Wallis rank sum test

c Pearson’s Chi-squared test

d Fisher’s Exact test

Significant p-values shown in bold.

**Table 2:** Incidence of Hypophosphatemia Across CKRT Fluid Types

Variable	Overall N = 823^a^	Type of CKRT Fluid			p-Value^b^
		Commercial PHOS+ N = 198^a^	Pharmacy compounded PHOS+ N = 374^a^	PHOS− N = 251^a^	
**Median phosphate level at CKRT initiation (mg/dl)**	4.7 (3.6–6.0)	4.6 (3.4–6.0)	4.5 (3.5–5.7)	5.1 (3.7–6.8)	**0.01**
**Median phosphate level during CKRT (mg/dl)**	3.9 (3.2–4.7)	3.9 (3.3–4.8)	3.8 (3.2–4.5)	3.8 (3.1–4.9)	0.50
**Minimum phosphate level during CKRT (mg/dl)**	2.9 (2.2–3.7)	3.1 (2.5–3.8)	2.8 (2.2–3.5)	3.0 (2.0–3.9)	0.06
**Hypophosphatemia (< 2.5 mg/dl)**	257 (31%)	46 (23%)	120 (32%)	91 (36%)	**0.01**

CKRT, Continuous kidney replacement therapy; PHOS+, Phosphate-containing; PHOS−, Phosphate-free.

a Median (Interquartile range)

b Kruskal-Wallis rank sum or Pearson’s Chi-squared test, as appropriate.

Significant p-values shown in bold.

**Table 3:** Univariable analysis for the associations of hypophosphatemia

Variable	Hypophosphatemia (< 2.5 mg/dl)		
	Yes N = 257^a^	No N = 566^a^	p-Value
**Age (years)**	8.8 (2.2–15.5)	9.0 (1.44–15.21)	0.30^b^
**Sepsis at ICU admission**	125 (49%)	235 (42%)	0.056^c^
**PELOD-2 score at CKRT initiation**	7 (5–9)	6 (4–9)	**0.037** ^b^
**%Fluid overload at CKRT initiation**	7.8 (2.6–18.1)	6.8 (2.0–16.5)	0.16^b^
**Mechanical ventilation at CKRT initiation**	181 (79%)	327 (74%)	0.10^c^
**CKRT Dose in mL/kg/hr**	43 (31–60)	42 (31–61)	0.81^b^
**CKRT duration (days)**	8 (5–15)	5 (2–15)	**<0.001** ^b^
**CKRT fluid type**			**0.011** ^c^
Commercially available PHOS+	46 (18%)	152 (27%)	
Pharmacy-compounded PHOS+	120 (47%)	254 (45%)	
PHOS−	91 (35%)	160 (28%)	

CKRT, Continuous kidney replacement therapy; ICU, Intensive care unit; PELOD, Pediatric logistic organ dysfunction; PHOS+, Phosphate-containing; PHOS−, Phosphate-free.

a Median (Interquartile range); n (%)

b Wilcoxon rank sum test

c Pearson’s Chi-squared test

Significant p-values shown in bold.

**Table 4:** Multivariable analysis for associations of hypophosphatemia

Variable	Reference	Contrast	Hypophosphatemia OR (95% CI)^a^	p-Value
**Age (years)**	1.7	15.3	1.01 (0.98–1.03)	0.56
**Sepsis at ICU admission**	No	Yes	1.23 (0.90–1.68)	0.20
**PELOD-2 score at CKRT initiation**	4	9	1.02 (0.98–1.07)	0.23
**CKRT fluid type**	PHOS−	Pharmacy-compounded PHOS+	0.85 (0.54–1.33)	0.47
**CKRT fluid type**	PHOS−	Commercially available PHOS+	0.62 (0.40–0.96)	**0.03**
**CKRT dose in mL/kg/hr**	31	60	1.0 (1.0–1.0)	0.89
**CKRT duration (days)**	3	15	1.0 (1.0–1.0)	0.74
**Center size**	Small	Medium	0.64 (0.43–0.95)	**0.03**
**Center size**	Small	Large	0.75 (0.46–1.21)	0.24

CKRT, Continuous kidney replacement therapy; ICU, Intensive care unit; PELOD, Pediatric logistic organ dysfunction; PHOS+, Phosphate-containing; PHOS−, Phosphate-free.

a Odds ratio (OR) and 95% confidence interval (CI) derived from logistic regression.

**Table 5:** Secondary outcomes for the entire cohort and divided by the presence of hypophosphatemia.

Variable	Overall N = 823^1^	Hypophosphatemia (< 2.5 mg/dl)		p-Value
		Yes N = 257^a^	No N = 566^a^	
**28-Day ventilator-free days**	3 (0–28)	1 (0–28)	0 (0–26)	0.08^b^
**28-Day ICU-free days**	0 (0–11)	0 (0–10)	0 (0–12)	0.90^b^
**28-Day hospital-free days**	0 (0–0)	0 (0–0)	0 (0–0)	0.35^b^
**ICU mortality**	300 (36%)	79 (31%)	221 (39%)	**0.02** ^3^
**90-Day mortality**	316 (38%)	86 (33%)	230 (41%)	**0.03** ^3^

ICU, Intensive care unit

a Median (Interquartile range); n(%)

b Wilcoxon rank sum test

c Pearson’s Chi-squared test

Significant p-values shown in bold.

**Table 6:** Multivariable analysis for risk factors of ICU mortality

Characteristic	OR	95% CI	p-Value
**Admission category**			**0.001**
Shock/infection/major trauma	1.26	0.77–2.07	
Respiratory failure	2.85	1.71–4.77	
Post-surgical/minor trauma	1.05	0.47–2.25	
Pain/sedation management	0.74	0.10–3.79	
Cardiac	1.72	0.96, 3.08	
**Sepsis at ICU admission**	1.12	0.77–1.62	0.55
**Presence of comorbidities**	3.14	1.99–5.08	**<0.001**
**PELOD-2 Score at CKRT initiation**	1.24	1.18–1.31	**<0.001**
**CKRT duration (days)**	1.00	1.00–1.01	0.41
**Hypophosphatemia**	0.58	0.30–1.07	0.082
**CKRT fluid type (reference = PHOS-)**			0.85
Commercially available PHOS+	1.01	0.60–1.70	
Pharmacy-compounded PHOS+	1.12	0.71–1.79	
**CKRT fluid type × hypophosphatemia** ^a^			0.29
Commercially available PHOS + × hypophosphatemia	1.74	0.65, 4.66	
Pharmacy-compounded × hypophosphatemia	0.83	0.37, 1.87	

CI, Confidence interval; CKRT, Continuous kidney replacement therapy; ICU, Intensive care unit; OR, Odds ratio; PELOD, Pediatric logistic organ dysfunction; PHOS+, Phosphate-containing; PHOS−, Phosphate-free.

a Model contains an interaction term between CKRT fluid type and hypophosphatemia.

Significant p-values shown in bold.

## WE-ROCK COLLABORATIVE AUTHOR LIST

The following individuals served as collaborators and investigators for WE-ROCK studies. They collaborated in protocol development and review, data analysis, and participated in drafting or review of the manuscript, and their names should be citable by PubMed.

Emily Ahern CPNP, DNP^1^, Ayse Akcan Arikan MD^2^, Issa Alhamoud MD^3^, Rashid Alobaidi MD, MSc^4^, Pilar Anton-Martin MD, PhD^5,6^, Shanthi S Balani MD^7^, Matthew Barhight MD, MS^8^, Abby Basalely MD, MS^9^, Amee M Bigelow MD, MS^10^, Gabriella Bottari MD^11^, Andrea Cappoli MD^11^, Abhishek Chakraborty MD^5^, Eileen A Ciccia MD^12^, Michaela Collins BA^13^, Denise Colosimo MD^14^, Gerard Cortina MD^15^, Mihaela A Damian MD, MPH^16,17^, Sara De la Mata Navazo MD^18^, Gabrielle DeAbreu MD^9^, Akash Deep MD^19^, Kathy L Ding BS^20^, Kristin J Dolan MD^2^, Lama Elbahlawan MD^21^, Sarah N Fernandez Lafever MD, PhD^18^, Dana Y Fuhrman DO, MS^13,22^, Ben Gelbart MBBS^23^, Katja M Gist DO MSc^1,20^, Stephen M Gorga MD, MSc^24^, Francesco Guzzi MD^25^, Isabella Guzzo MD^11^, Taiki Haga MD^26^, Elizabeth Harvey MD^27^, Denise C Hasson MD^28^, Taylor Hill-Horowitz BS^9^, Haleigh Inthavong BS, MS^2^, Catherine Joseph MD^2^, Ahmad Kaddourah MD, MS^29^, Aadil Kakajiwala MD, MSCI^30^, Aaron D Kessel MD, MS^9^, Sarah Korn DO^31^, Kelli A Krallman BSN, MS^13^, David M Kwiatkowski MD Msc^16^, Jasmine Lee MSc^27^, Laurance Lequier MD^4^, Tina Madani Kia BS^4^, Kenneth E Mah MD, MS^16,32^, Eleonora Marinari MD^11^, Susan D Martin MD^33^, Shina Menon MD^16,34^, Tahagod H Mohamed MD^10^, Catherine Morgan MD MSc^4^, Theresa A Mottes APRN^8^, Melissa A Muff-Luett MD^35^, Siva Namachivayam MBBS^23^, Tara M Neumayr MD^12^, Jennifer Nhan MD, MS^7,30^, Abigail O’Rourke MD^9^, Nicholas J Ollberding PhD^13^, Matthew G Pinto MD^36^, Dua Qutob MD^29^, Valeria Raggi MD^11^, Stephanie Reynaud MD^37^, Zaccaria Ricci MD^14^, Zachary A Rumlow DO^3,24^, María J Santiago Lozano MD, PhD^18^, Emily See MBBS^23^, David T Selewski MD, MSCR^38,39^, Carmela Serpe MSc, PhD^11^, Alyssa Serratore RN, MsC^23^, Ananya Shah BS^20^, Weiwen V Shih MD^1,20^, H Stella Shin MD^17^, Cara L Slagle MD^40^, Sonia Solomon DO^36,41^, Danielle E Soranno MD^40^, Rachana Srivastava MD^43^, Natalja L Stanski MD^13^, Michelle C Starr MD, MPH^40^, Erin K Stenson MD^1,20^, Amy E Strong MD, MSCE^3^, Susan A Taylor MSc^19^, Sameer V Thadani MD^2^, Amanda M Uber DO^35,42^, Brynna Van Wyk ARNP, MSN^3^, Tennille N Webb MD, MSPH^44^, Huaiyu Zang PhD^13^, Emily E Zangla DO^7^, Michael Zappitelli MD, MSc^27^

^1^Children’s Hospital Colorado, Aurora, CO, USA

^2^Baylor College of Medicine, Texas Children’s Hospital, Houston, TX, USA

^3^University of Iowa Stead Family Children’s Hospital, Carver College of Medicine, Iowa City, IA, USA

^4^Univeristy of Alberta, Edmonton, Canada

^5^Le Bonheur Children’s Hospital, Memphis, TN, USA

^6^Children’s Hospital of Philadelphia, University of Pennsylvania Perelman School of Medicine, PA, USA

^7^University of Minnesota, Minneapolis, MN, USA

^8^Ann and Robert H. Lurie Children’s Hospital of Chicago, Chicago, IL, USA

^9^Cohen Children’s Medical Center, Zucker School of Medicine, New Hyde Park, NY, USA

^10^Nationwide Children’s Hospital, The Ohio State University College of Medicine, Columbus, OH, USA

^11^Bambino Gesù Children’s Hospital, IRCCS, Rome, Italy

^12^Washington University School of Medicine, St. Louis Children’s Hospital, St. Louis, MO, USA

^13^Cincinnati Children’s Hospital Medical Center; University of Cincinnati College of Medicine, Cincinnati, OH, USA

^14^Meyer Children’s Hospital, IRCCS, Florence, Italy

^15^Medical University of Innsbruck, Innsbruck, Austria

^16^Stanford University School of Medicine, Palo Alto, CA, USA

^17^Children’s Healthcare of Atlanta, Emory University School of Medicine, Atlanta, GA, USA

^18^Gregorio Marañón University Hospital; School of Medicine, Madrid, Spain

^19^King’s College Hospital, London, England

^20^University of Colorado, School of Medicine, Aurora, CO, USA

^21^St Jude Children’s Hospital, Memphis TN USA

^22^University of Pittsburgh Medical Center Children’s Hospital of Pittsburgh, Pittsburgh, PA, USA

^23^Royal Children’s Hospital, University of Melbourne, Murdoch Children’s Research Institute, Melbourne, Victoria, Australia

^24^University of Michigan Medical School, C.S. Mott Children’s Hospital, Ann Arbor, MI, USA

^25^Santo Stefano Hospital, Prato, Italy

^26^ Osaka City General Hospital, Osaka, Japan

^27^Hospital for Sick Children, Toronto, Ontario, Canada

^28^NYU Langone Health, Hassenfeld Children’s Hospital, New York, NY, USA

^29^ Sidra Medicine and Weil Cornel Medicine, Qatar, Doha, Qatar

^30^Children’s National Hospital, Washington, DC, USA

^31^Westchester Medical Center, Westchester, NY, USA

^32^University of California, San Francisco, CA, USA

^33^Golisano Children’s Hospital at University of Rochester Medical Center, Rochester, NY, USA

^34^Seattle Children’s Hospital, University of Washington, Seattle, WA, USA

^35^University of Nebraska Medical Center, Children’s Hospital & Medical Center, Omaha, NE, USA

^36^Department of Pediatrics, Westchester Medical center, Maria Fareri Children’s, Valhalla, NY, USA

^37^Dalhouse University, Halifax, Nova Scotia, Canada

^38^Medical University of South Carolina, Charleston, SC, USA

^39^Arkansas Children’s Hospital, University of Arkansas for Medical Sciences, Little Rock, AR, USA

^40^Indiana University School of Medicine, Riley Hospital for Children, Indianapolis, IN, USA

^41^Department of Pediatrics, Nemours Children’s Hospital, Wilmington DE, USA

^42^University of Utah School of Medicine, Primary Children’s Hospital, Salt Lake City, UU, USA

^43^Mattel Children’s Hospital at UCLA, Los Angeles, Ca, USA

^44^Children’s of Alabama/University of Alabama at Birmingham, Birmingham, AL, USA,

## Abbreviations

AKI: acute kidney injury
CI: confidence interval
CKRT: continuous kidney replacement therapy
ICU: intensive care unit
IQR: interquartile range
IRB: institutional review board
OR: odds ratio
PELOD-2: Pediatric Logistic Organ Dysfunction score
PHOS+: phosphate-containing (CKRT fluid)
PHOS−: phosphate-free (CKRT fluid)
WE-ROCK: Worldwide Exploration of Renal Replacement Outcomes Collaborative in Kidney Disease
